# Phase 1 safety trial of a natural product cocktail with antibacterial activity in human volunteers

**DOI:** 10.1038/s41598-022-22700-4

**Published:** 2022-11-16

**Authors:** Julie Bruce, Blessing Oyedemi, Nick Parsons, Freya Harrison

**Affiliations:** 1 Warwick Clinical Trials Unit, Division of Health Sciences, University of Warwick, Coventry, CV4 7AL UK; 2 University Hospital Coventry and Warwickshire NHS Trust, Clifford Bridge Road, Coventry, CV2 2DX UK; 3 School of Life Sciences, University of Warwick, Coventry, CV4 7AL UK; 4 Statistics and Epidemiology Unit, Warwick Medical School, University of Warwick, Coventry, CV4 7AL UK

**Keywords:** Microbiology, Health care, Medical research

## Abstract

New antibiotics are urgently needed to reduce the health burden of antibiotic-resistant bacterial infection. Natural products (NPs) derived from plants and animals are a current focus of research seeking to discover new antibacterial molecules with clinical potential. A cocktail of NPs based on a medieval remedy for eye infection eliminated biofilms of several highly antibiotic-resistant bacterial species in laboratory studies, and had a promising safety profile in vitro and in a mouse model. A necessary prelude to refining this remedy into a defined, synthetic mixture suitable for testing with wound infections is to firstly establish safety when applied to healthy human skin. We aimed to assess skin-related outcomes of the preparation in a sample of healthy volunteers. This prospective, single arm, non-randomised Phase I clinical trial consisted of a single patch test intervention with 48-h follow-up. Volunteers were staff, students and members of the public recruited from the University of Warwick and surrounding locality. Adults aged 18–79 years, with no history of severe immunity-related disease, diabetes, recent infection, or known pregnancy were eligible. A 100 µl application of a filter-sterilised NP mixture, comprising ground garlic, onion, white wine and bovine bile, was applied to skin on the upper arm and covered with a dressing. The primary outcome was skin-related adverse events over 48 h. Digital photographs were captured where bothersome, salve-related events were reported. 109 volunteers, aged 18–77 years, were recruited between June and July 2021. Sample mean age was 37.6 (SD 16.1) years, and 63 (58%) participants were female. Outcome data were obtained for 106/109 (97%); two participants were lost to follow-up and one removed the skin patch after nine hours due to a bothersome garlic odour. Twenty-one (19.8%) participants reported any patch-test related sign or symptom; of these 14 (13.2%) participants reported minor events related to the salve, including itchiness, redness, or garlic odour. No serious events were reported. We found no evidence of serious skin-related adverse events related to the NP preparation.

*Trial registration*: International Standard Randomised Controlled Trial Number (ISRCTN10773579). Date registered: 08/01/2021.

## Introduction

Antimicrobial resistance is a global health emergency^1,2^. Widespread antibiotic use has led to bacteria evolving various genetic strategies to develop drug resistance, and the alarming spread of multidrug resistant infections continues to compromise the ability of available antibiotics to treat common infections. The scarcity of new antibacterial drugs in later stages of development increases the risk of untreatable infections, especially those caused by Gram-negative bacteria^3,4^. Wound and soft-tissue infections caused by bacteria that form biofilms—aggregated, multicellular communities of bacteria protected by a self-produced slime matrix—are particularly refractory to antibiotic treatment and pose a serious clinical and economic concern^5,6^. Biofilm infections of surgical or trauma wounds, pressure sores, venous ulcers and diabetic foot ulcers can be multiply antibiotic resistant and become chronic, lasting for months or years. These infections are expensive to manage and may lead to sepsis; preventing progression to sepsis may require radical, life-altering intervention, such as limb amputation^7–9^.

Most currently deployed antibiotics are derived from fungal or bacterial secondary metabolites. However, in recent years there has been an increasing recognition that refilling the antibacterial drug discovery pipeline requires researchers to cast the net wider in the hunt for antibacterial and anti-biofilm molecules^10,11^. Promising alternatives to “traditional” antibiotics include a wide range of plant- and animal-derived natural products. Preparations of mānuka (*Leptospermum* sp.) honey incorporated into ointments or advanced wound care dressings are widely used, as honey has proven antimicrobial activity and may accelerate wound healing^12,13^. Antimicrobial peptides produced by a range of organisms are the subject of numerous clinical and preclinical trials^14,15^. Acetic acid (the acid present in vinegar) is used as an antibacterial agent to treat burn-related wound infections^16,17^. Further, glycoside hydrolase enzymes are being formulated into experimental wound treatments as they have potential to break down biofilm matrix polysaccharides and facilitate entry of antibiotics into the biofilm^18,19^.

Often, the rationale for exploring particular NPs for drug development stems from their use in traditional or historical medicine. Perhaps the most famous example of this is the anti-malarial drug artemisinin, isolated from sweet wormwood (*Artermisia annua*), following directions for medicinal use in a fourth century Chinese text^20^. The tenth century English medical text known as Bald’s Leechbook (British Library Royal MS 12 D XVII) contains a similar remedy for seasonal malaria, using native *Artemisia* species^21^. Whilst historical European infection remedies have been less well studied from an ethnopharmacological perspective than their counterparts in Asian African and South American traditional medicine, pre-modern European medical texts contain a range of treatments for symptoms of microbial infection which contain NPs known to have some antimicrobial activity (e.g. honey, vinegar and various plant species)^22–25^.

Our team has extensively tested a remedy for eye infection from Bald’s Leechbook for its potential to kill pathogenic bacteria grown as biofilms in conditions mimicking soft-tissue chronic wounds. We demonstrated that a reconstruction of this NP cocktail, known as Bald’s eyesalve, possesses remarkable ability to kill biofilms of *Staphylococcus aureus*, *Acinetobacter baumannii* or *Streptococcus pyogenes* in an ex vivo wound model, and to kill bacteria in biofilms of methicillin-resistant *S. aureus* in biopsies taken from mouse chronic wound infections^26,27^. The eyesalve can kill other Gram-positive and Gram-negative wound pathogens when grown planktonically^26^. It may thus have capacity to aid in biofilm clearance of these species when combined with antibiotics or adjuvants that enhance penetration through the biofilm matrix. Bald’s eyesalve is a preparation of garlic (*Allium sativum*), onion (*A. cepa*), wine and bovine bile, and its antibiofilm efficacy relies on the combination of all these constituents^26,27^. Efficacy cannot be attributed to any single ingredient in the cocktail, i.e. the combination and preparation of ingredients generates a cocktail of compounds and/or a novel compound which effectively kills bacteria. A recent study from our laboratory showed that Bald’s eyesalve possess a promising safety profile in vitro, in an ex vivo model of eye irritation, and in a mouse model of chronic wound healing^28^. Garlic can cause severe irritation and burns when applied to skin^29^, but our mouse experiments suggested that this effect may be ameliorated when garlic is prepared along with other ingredients, following the medieval eyesalve instructions.

Our research team is currently conducting chemical fractionation and analysis of Bald’s eyesalve in order to identify which compounds in the mixture are responsible for its antibacterial activity. Our ultimate aim is to develop a defined, synthetic mixture of only those compounds which are responsible for antibiofilm activity (molecules which confer or potentiate antibacterial activity). Such a synthetic mixture would be suitable for incorporation into an advanced wound dressing, which could then be subjected to clinical testing for efficacy against wound infections. But a necessary step in this process is to determine whether the promising safety profile of the original mixture that we found in laboratory tests, translates to a promising safety profile when applied to human skin. If the original mixture causes significant irritation, then this would cast doubt on the utility of further labour-intensive and expensive development of a synthetic derivative.

We aimed to assess the safety of Bald’s eyesalve when applied to healthy human skin, by undertaking a patch test in a sample of healthy volunteers to investigate incidence of adverse events over 48 h. As raw garlic can cause pain within a few hours when applied directly to skin and covered with a dressing, we reasoned that 48-h would be a conservative window to observe any adverse events. Our null hypothesis was that 48-h exposure to the ancientbiotic would not result in clinically significant skin irritation or inflammation, or any serious adverse effects, in healthy, non-diabetic and non-allergic adults. We believe this is the first time that a medieval “ancientbiotic” comprising multiple ingredients has been assessed in a Phase I trial with healthy volunteers.

## Results

**Figure 1:**
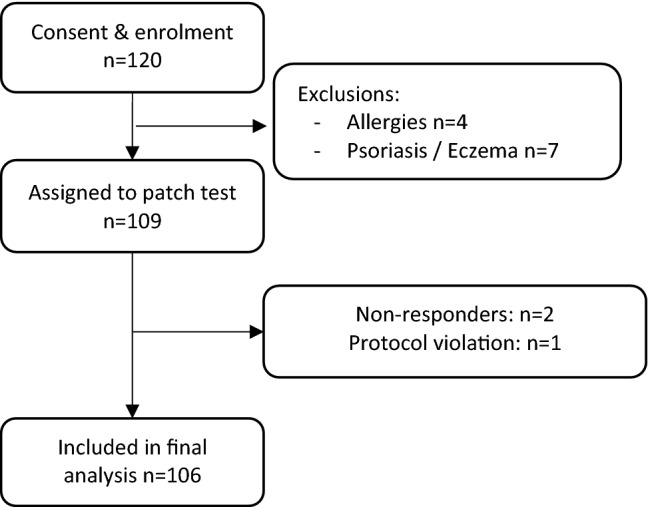
Trial flowchart.

We recruited 109 participants, aged 18–77 years, between 1 June 2021 and 21 July 2021. We recruited the sample in phases, firstly recruiting five volunteers, to pilot data collection forms and to ensure no serious adverse event occurred within 48 h of salve application. We then proceeded to recruit the remaining sample. Figure 1 presents participant flow as per CONSORT. Sample mean age was 37.6 (SD 16.1) years, and 63 (58%) participants were female. Summary demographic data are presented in Table 1. Outcome data were obtained for 106 participants (97%; 106/109): two participants were lost to follow-up and one participant removed the skin patch after nine hours due to bothersome garlic odour (deemed a protocol violation).

**Table 1:** Participant characteristics.

	Total recruited
	N = 109
**Gender, n (%)**	
Female	63 (57.8)
Male	45 (41.3)
Non-binary	1 (0.9)
**Age, years**	
Mean (SD)	37.6 (16.1)
Median (range)	31 (18, 77)
**Eyesalve batch applied**	
Batch number 1	57 (52.3)
Batch number 2	52 (47.7)

A total of 21 (19.8%; 21/106) participants providing outcome data reported at least one adverse sign or symptom; of these 14 were salve-related and seven related only to the dressing applied over the skin-patch. This was verified using the participants’ own reports and by digital photographs: Figure S1a and S1b show a mild skin reaction to the dressing adhesive and a skin reaction to the eye-salve respectively.

Seven participants reported one adverse reaction to the salve: either minor redness (n = 2), slight itching (n = 3), mild discomfort (n = 1) or dry skin (n = 1) (Table 2). One participant reported multiple symptoms of itchiness, redness and spreading redness on the upper arm, although this was described as short-lasting. Finally, one participant reported itching, discomfort, heat and broken skin; we deemed this a clinically important reaction to the salve although the participant did not provide a digital image. All other events were described by respondents as either minor and/or transitory. We did not set out to explicitly test for effects of gender, age or salve batch on AEs, but our data did not show any apparent skew in incidence of events by any of these variables (Table S1, Figure S2).

**Table 2:** Signs and symptoms reported by 106 participants who provided outcome data at 48 h.

	N = 106
	N (%)
**Signs and symptoms**	
Any	21 (19.8)
Salve-related only	14 (13.2)
Dressing-related only*	7 (6.6)
**Specific salve-related AEs**	
Redness at site	7 (6.6)
Spreading redness	1 (0.9)
Itchiness	8 (7.5)
Discomfort	4 (3.8)
Heat	1 (0.9)
Pain	0 (0.0)
Broken skin	1 (0.9)
Burning/tingling	1 (0.9)
Dry skin	1 (0.9)
Garlic odour	1 (0.9)
**N symptoms attributable to the salve**	
0	92 (87.8)
1	7 (6.6)
2	5 (4.7)
3–5	2 (1.9)

## Discussion

Antimicrobial resistance is a major threat to human health and there is an urgent need to restock the antibiotic development pipeline. Of particular concern are chronic wound infections caused by bacterial biofilms: these are debilitating, costly to treat and can prove fatal. Natural products provide a potential pool of candidate compounds for development into clinically-useful antibacterial agents^11,30^. Historical medical texts can act as a database of NPs which have traditionally been used to treat infection, with a rich set of contextual data on how plants and other natural materials were prepared, combined and assigned to treat symptoms of conditions that we know to be microbial infections^22,23,31^.

We investigated the safety profile of a medieval “ancientbiotic”, a preparation of four natural ingredients which we found to have excellent antibacterial and antibiofilm activity in laboratory and animal models. Our previous work suggested a promising safety profile in an ex vivo model of eye irritation and a mouse model of wound healing^26–28^. This trial represents the first topical application of our reconstructed remedy to human participants. Overall, we found the salve was well tolerated by healthy participants, including by a small sample of older participants whose skin may be more sensitive to irritants. Only one person reported an adverse reaction, describing localised broken skin along with localised redness, itchiness, discomfort and heat. Other recorded AEs were described as minor and short-lasting. The salve was not associated with any moderate to severe skin-related adverse events. Where adverse reactions were recorded and attributed to the salve, rather than to the adhesive of the dressings used, these were typically described as mild. Only one participant removed their skin patch as they found the garlic odour unpleasant, causing a headache. While considerations of odour are understandably important to healthy people, this may be less of a concern for those living with chronic infected wounds; these are often malodorous, and the health risks of a non-resolving wound infection are severe. Further research will be required to explore the acceptability of wound dressings impregnated with odorous compounds derived from garlic.

Demand for antimicrobial dressings for the treatment of chronic wounds and related skin ulcer complications is increasing in the wound care market. The most common “traditional” antibiotics used in antimicrobial dressings are bacitracin A, neomycin, fucidin, mupirocin and retapamulin. Due to the emergence of antibiotic-resistant bacteria, researchers, companies and clinicians are shifting focus to antibiotic alternatives. Innovations in antimicrobial dressing technology which show effective pathogen killing but low harm to healthy skin include silver, honey, iodine, acetic acid and others. Silver dressings account for 70% of the global antimicrobial dressings segment and are the most preferred antimicrobial dressings in high-income countries. They are, however, expensive and many companies are seeking alternatives to silver. Honey-based dressing are also popular and are gaining attention in lower-income countries as they have a lower cost. Wound care products derived from plant materials are also on the market, including films, hydrofibre, hydrocolloids, collagen, alginates, surfactants (e.g. Braun Medical Inc.’s Prontosan Wound Gel X which contains betaine) and foams (e.g. ConvaTec’s silicone Foam Lite dressing). Thus, there is a potential market for an advanced antimicrobial wound dressing that relies on natural products to manage infection.

Most molecules that have promising ability to kill bacteria in simple planktonic culture in the laboratory ultimately fail to show clinical potential as antibacterial agents, and many antibacterial agents that have good ability to kill bacteria in simple, acute infections have reduced or no activity against bacteria growing as biofilms^5,6^. Thus, the fact that the salve shows such strong potential to kill bacteria growing as biofilms in high-validity lab models of wound biofilm infection makes it a worthy candidate for further research. Developing the raw mixture of ingredients into a defined cocktail of purified natural molecules with a defined mechanism of action will be essential to formulate a product that would be amenable to accurate dosing and reproducible, scalable manufacture. This work will also allow us to determine whether odour can be reduced while retaining efficacy. This will require significant effort in analytical chemistry and microbiology. Thus, one key stop/go point before such an investment is to assess whether the raw preparation is relatively safe for human use. If the raw preparation had triggered significant or severe adverse reactions, it would be highly unlikely that molecular cocktail development could progress.

### Limitations

We acknowledge the risk of selection bias. Our inclusion criteria were applied to accept only healthy volunteers at low risk of developing a skin reaction. We cannot, therefore, generalise findings to other populations with significant comorbidity. The salve may cause irritation on those with broken skin or open wounds. Patients with, or who are at risk of, non-healing wounds will generally have underlying characteristics that make them more sensitive to skin irritation or inflammation. Diabetic patients can have dysregulated immune responses and may also have painful neuropathy which will impact upon symptom reporting^32^. A further limitation was the number of minor reactions to the adhesive on the hypoallergic covering dressings, which in some cases made it more challenging to determine whether an event was attributable to the salve or the protective dressing. Assessments were undertaken by a trained healthcare professional within the research team rather than by an independent clinician or dermatologist. We have previous experience of judging adverse skin outcomes, including redness, spreading erythema, exudate and wound healing, in other patient populations, where inter-rater reliability was found to be substantial^33^.

A test in healthy participants is a necessary prerequisite before testing the salve with a hospitalised or community-based sample of patients with chronic wounds. Taken together with our published observation that the salve did not interfere with normal healing in experimentally wounded mice^28^, our results provide some justification for a future trial with a participant group who better reflect the age and health profile of people at risk of chronic wounds. A future trial should test the salve using a surgical dressing or advanced wound care dressing with known tolerability e.g. post-operative woven or alginate dressing typically used in chronic wound management. As chronic wound care typically involves extended periods of dressing use, future work should also extend the study period to detect adverse events over a longer observation period.

## Conclusions

The reconstructed medieval ancientbiotic was well tolerated and had a good safety profile when applied to healthy human skin over a 48-h exposure period. There were no serious adverse events amongst our sample of healthy volunteers. Our findings suggest that further molecular investigation and testing of Bald’s eyesalve is worthwhile.

## Methods

### Study design

Non-randomised, non-controlled, open-label Phase I trial. Due to the odour associated with the garlic content in the salve, and the need to fully inform participants of salve ingredients and to exclude those with known allergies, it was not possible to blind participants to treatment allocation. Randomisation was not undertaken as no control arm was included at this stage of development.

### Participants

We aimed to recruit 100 community-dwelling male and female volunteers, aged from 18 to 79 years. People were eligible if they self-reported as having no diagnosis of diabetes or severe immune-related disease (allergic asthma, eczema, psoriasis), had no known allergy to any of the salve ingredients, and they could comply with the study protocol. Those known to be pregnant were excluded, as were people with broken skin at the site of salve application. People with any recent infection, including Coronavirus Disease-2019 (Covid-19), caused by the Severe Acute Respiratory Syndrome CoronaVirus-2 (SARS-CoV-2), or with any suspected symptoms of Covid-19, or who were self-isolating due to Covid-19, were excluded. Adults unable to read English and those lacking capacity to consent were excluded.

### Study procedures

#### Screening and recruitment

Volunteers were identified using multiple strategies, including email circular invitations to staff and students at the University of Warwick during academic term time and advertisements on social media (Twitter). We advertised through local community groups e.g. Women’s Institute, walking groups, to attract a wider age range. Volunteers expressing interest were given study information and invited to attend a short clinic appointment at Warwick Medical School.

#### Intervention preparation

A fresh batch of Bald’s eyesalve was prepared following our published protocol^26–28^. We used garlic and onions sourced from a commercial greengrocer, Pennard Organic white wine (11% ABV; Avalon Vineyard, Somerset), and dried bovine bile (Merck Millipore catalogue no. B3883, suspended in sterile water to a concentration of 89 mg/ml). After preparation, the salve was passed through a 0.2 µm syringe filter to ensure sterility and aliquots stored at − 20 °C in plastic microfuge tubes until needed. Two independent batches of the salve were prepared, using fresh ingredients each time. To assess the activity of the eyesalve batches against a bacterial biofilm, fifteen biofilms of *Staphylococcus aureus* Newman were cultured in a collagen-based synthetic wound model for 24 h at 37 °C. The mature biofilms were then topically treated with the eyesalve (n = 5 per batch) or with sterile water (n = 5) as a control and incubated for a further 24 h at 37 °C. The synthetic wound matrix was then degraded by treating with collagenase, and bacteria recovered for enumeration by plating. Both batches of eyesalve reduced the number of viable bacteria by 7–8 log_10_ fold, and thus are among the most active batches of eyesalve typically prepared by our laboratory (See Fig. 2 in ref^26^). A full description of the biofilm testing methods, and the full results, are provided in the Supplementary Information.

#### Patch test application

A total of 100 µl of Bald’s eyesalve was applied onto the absorbent pad of a sticking plaster (HypaPlast 7.2 × 2.5 cm hypoallergenic Blue Detectable Plaster, Safety First Aid Group Ltd, UK) then applied to the participant’s upper arm. This was covered with a larger dressing to ensure the site remained waterproof (Primapore 8.3 × 6 cm dressing, Smith and Nephew, UK). Participants were instructed to wear the patch for 48 h. Due to Covid-19, clinics were held outdoors, to maximise ventilation and adhere to local social distancing guidance. Face coverings were worn by study personnel and participants, and disposable medical gloves were worn by study personnel and replaced after each application. Study personnel and participants sanitised their hands before and after touching any study materials.

#### Outcomes

We designed data collection forms to record skin-related outcomes and other adverse events at 48 h. We recorded binary outcomes for any sign of redness at the application site; redness spreading beyond the patch test area; itchiness; discomfort near or around the patch test area; heat near or around the patch test area; pain near or around the patch test area; any signs of broken skin; and any other suspected adverse reaction. Free-text descriptions of reactions were recorded. Participants were contacted face-to-face, or by telephone, text message, email or video call (Microsoft Teams, Zoom), according to the participant’s expressed communication preference, for outcome data collection. Digital photographs were requested from participants describing a bothersome skin reaction. Judgement of skin reactions was undertaken by an experienced healthcare researcher trained in skin allergy testing (JB).

#### Adverse event management

A safety reporting protocol was developed for related and unexpected adverse events (AEs) and serious adverse events. An AE was defined as any untoward medical occurrence which did not necessarily have a causal relationship with the intervention. The study was administered in keeping with Standard Operating Procedures for risk assessment and monitoring at Warwick Clinical Trials Unit.

#### Risk assessment

Although the salve incorporated edible household ingredients (except for bile), we anticipated some risk of skin redness or irritation from the patch test, either from the salve or sticking plaster. We did not anticipate any serious adverse events resulting in death, hospitalisation, life threatening illness or permanent disability. Any participant experiencing any skin discomfort was advised to remove the plaster and gently wash the area with soap and water. Where symptoms persisted, they were advised to visit their general practitioner.

#### Withdrawals

Participants could opt to withdraw from the trial before treatment started or after the salve was applied, for any reason, without giving reason.

### Sample size

This was a phase I safety study that aimed to identify any acute skin reactions caused by the ancientbiotic salve, added onto the absorbent part of the sticking plaster applied to the upper arm. We anticipated that the plaster itself could cause some minor skin irritation, independent of the salve, therefore we estimated that between 10 and 20% of participants may report some symptoms. To facilitate planning of the follow-on (phase II/III) studies, we aimed to estimate event rates with reasonable precision. This was achieved by aiming to recruit a sample size of 100 participants which would allow us to estimate rates with 95% confidence intervals (CIs) with (approximate) widths, at the extremes of the anticipated range, of 5–20% at 10% and 15–30% at 20%. A sample size of 100 participants also allowed us to capture a distribution of adverse skin effects across a wider age distribution.

### Statistical analysis

Type, number (%) and distribution of adverse skin reactions were recorded. Continuous data were summarised using mean, standard deviation, median and range values. We report findings following CONSORT recommendations^34^. Data were recorded in Microsoft Excel and statistical analyses were undertaken on a complete case basis, performed in R version 4.0.4^35^. We did not plan imputation analyses as data missingness was expected to be low, due to the short follow-up period and strong participant engagement.

### Data security and management

Study participant data were stored on a secure database in accordance with the UK General Data Protection Regulation (UK GDPR), tailored by the Data Protection Act 2018. A unique trial identification number was used for participant communication. All clinical forms were checked for completeness before data entry then double checked by two research team members independently. Trial documentation will be archived for at least ten years in accordance with Warwick Clinical Trials Unit standard operating procedures. We confirm that all methods were performed in accordance with recommended guidance and regulations for studies with human participants, in accordance with the Declaration of Helsinki.

### Development of patient materials/public involvement

Development of the grant application which led to the work being funded was informed by feedback from a small focus group of people with diabetes. The proposed laboratory work and clinical trial was reviewed by a panel of people with diabetes and carers of people living with diabetes as part of the funder’s standard procedures. The Quality Assurance team at Warwick Clinical Trials Unit contributed to data collection materials and advised on recruitment-related issues.

### Role of funder

The study was funded by Diabetes UK, under Project Grant ref. 17_0005690. The funder had no role in data collection, data interpretation or writing of the report.

### Ethics approval

Ethical approval was granted from the University of Warwick Biomedical and Scientific Research Ethics Committee (04/11/2020; Ref: 03/20-21). Volunteers were provided with details of the salve ingredients and given sufficient time to consider the patient information sheet and ask questions. Eligibility checks and written informed consent was taken in person by a member of the research team (BO/FH/JB). We sought consent to obtain and store digital images of skin-related adverse reactions. The trial was listed on a public research registry (08/01/2021; ISRCTN10773579).

## Supplementary Information

Supplementary Information 1.

Supplementary Information 2.

## Data Availability

Requests for data should be sent to the corresponding authors. Access to anonymised data may be granted following review.

## Abbreviations

AE: Adverse event
CONSORT: Consolidated Standards of Reporting Trials
NP: Natural product
