# Supplementary material for: Phase 1 safety trial of a natural product cocktail with antibacterial activity in human volunteers
Source: Sci Rep. 2022 Nov 16;12:19656. doi: 10.1038/s41598-022-22700-4 (PMC9667429; doi:10.1038/s41598-022-22700-4)
Supplement: Supplementary file 1 — Supplementary Information 1. [file 41598_2022_22700_MOESM1_ESM.docx]

**Supplementary Information, file 1**

**Phase 1 safety trial of a natural product cocktail with antibacterial activity in human volunteers**

**Table S1.** Incidence of salve-related adverse effects by (a) gender; (b) batch number used.

| **a)** | Female N | Male N | Non-binary N | Total |
| --- | --- | --- | --- | --- |
| No AE | 52 | 39 | 1 | *92* |
| ≥1 AE | 9 | 5 | 0 | *14* |
| Total | 61 | 44 | 1 | **106** |
|  |  |  |  |  |
| **b)** | Batch 1 | Batch 2 | Total |  |
| No AE | 49 | 43 | *92* | - |
| ≥1 AE | 6 | 8 | *14* | - |
| Total | 54 | 51 | **106** | - |

**Figure S1.** Examples of (a) a skin reaction to the dressing adhesive only and (b) a skin reaction to the eyesalve

**Figure S1a. Figure S1b.**


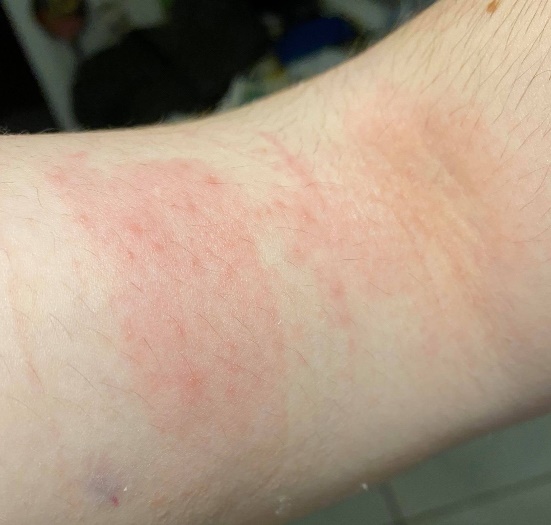
**
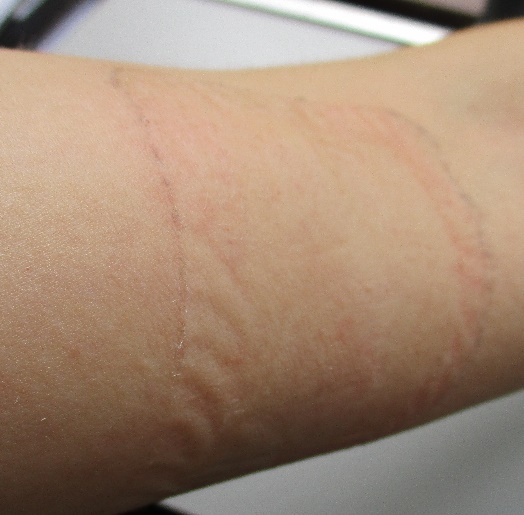
**

**Figure S2.** Median (inter-quartile) age distribution of participants by salve-related AEs
