# Supplementary material for: Phase 1 safety trial of a natural product cocktail with antibacterial activity in human volunteers
Source: Sci Rep. 2022 Nov 16;12:19656. doi: 10.1038/s41598-022-22700-4 (PMC9667429; doi:10.1038/s41598-022-22700-4)
Supplement: Supplementary file 2 — Supplementary Information 2. [file 41598_2022_22700_MOESM2_ESM.docx]

**Supplementary file 2**

**Phase 1 safety trial of a natural product cocktail with antibacterial activity in human volunteers**

**Julie Bruce^1^, Blessing Oyedemi^2^, Nick Parsons^2^, Freya Harrison^1^.**

***Supplementary Information: Testing of the eyesalve batches for antibiofilm activity***

Biofilms were created in a synthetic soft-tissue wound model as described by Werthén et al*.* [1] and as used in our previous work [2-4]. Briefly, synthetic wounds were created on ice and comprised 2 mg·ml^−1^ collagen, 0.01% acetic acid, 60% [vol/vol] synthetic wound fluid SWF: a 1:1 mixture of foetal bovine serum (Gibco) and peptone water (Sigma-Aldrich), and 10 mM sodium hydroxide. 200µl aliquots of this mixture were dispensed into the wells of 48-well culture plate, placed under short-wave UV light for ten minutes to sterilise the wounds, and then incubated at 37 °C for 1 h to allow collagen to fully polymerise.

*Staphylococcus aureus* (Newman) was grown aerobically overnight at 37 °C on an LB agar plate and colonies transferred to 5ml SWF; this inoculum was incubated at 37 °C on an orbital shaker for 6 hours (mid log phase). This starter culture was diluted to an OD_600_ of 0.05-1 in fresh SWF, and 50µl of this suspension was added to each freshly-made synthetic wound. Inoculated wounds were incubated at 37 °C for 24 h to allow biofilm formation.

Wounds containing mature biofilms were then exposed to 100 µl Bald’s eyesalve (Batch 1, n=5 or batch 2, n=5) or 100µl sterile water (n=5) and incubated at 37 °C for a further 24 h. Bacteria were recovered by treating with 300 μl of 0.5 mg·ml^−1^ collagenase type 1 (EMD Millipore Corp, USA) for 1 h at 37 °C to break down the collagen matrix; the resulting liquid was serially diluted and plated on LB agar plates. Plates were incubated at 37 °C overnight, colonies were counted, and colony-forming units (CFU) per wound calculated to give an estimate of the viable bacterial cell count in each wound.

The data obtained are provided below. The detection threshold for this experiment (the lowest number of viable bacteria that could be detected in the wounds using our dilution and plating strategy) was 22 – when no colonies were observed for a wound, this value is entered into the table.

| **Treatment** | **Replicate** | **Total CFU** |
| --- | --- | --- |
| Control | 1 | 1.17E+09 |
| Control | 2 | 1.02E+09 |
| Control | 3 | 1.02E+09 |
| Control | 4 | 6.38E+08 |
| Control | 5 | 8.94E+08 |
| Eyesalve batch 1 | 1 | *22* |
| Eyesalve batch 1 | 2 | *22* |
| Eyesalve batch 1 | 3 | *22* |
| Eyesalve batch 1 | 4 | *22* |
| Eyesalve batch 1 | 5 | *22* |
| Eyesalve batch 2 | 1 | *22* |
| Eyesalve batch 2 | 2 | *22* |
| Eyesalve batch 2 | 3 | *22* |
| Eyesalve batch 2 | 4 | *22* |
| Eyesalve batch 2 | 5 | *22* |

[1] Werthén M, Henriksson L, Jensen PØ, Sternberg C, Givskov M, Bjarnsholt T. An in vitro model of bacterial infections in wounds and other soft tissues. *APMIS*. 2010;118(2):156-64. 10.1111/j.1600-0463.2009.02580.x

[2] Furner-Pardoe J, Anonye BO, Cain R, Moat J, Ortori CA, Lee C, et al. Anti-biofilm efficacy of a medieval treatment for bacterial infection requires the combination of multiple ingredients. Scientific Reports. 2020;10(1):12687.

[3] Harrison F, Roberts AEL, Gabrilska R, Rumbaugh KP, Lee C, Diggle SP. A 1,000-year-old antimicrobial remedy with antistaphylococcal activity. mBio. 2015;6(4):e01129-15.

[4] Anonye BO, Nweke V, Furner-Pardoe J, Gabrilska R, Rafiq A, Ukachukwu F, et al. The safety profile of Bald’s eyesalve for the treatment of bacterial infections. Scientific Reports. 2020;10(1):17513.
